# The Combined Effects of EEG Biofeedback and Olanzapine on Glucose and Lipid Metabolism, Cardiac Function, and Cognitive Function in Patients With Schizophrenia

**DOI:** 10.62641/aep.v53i3.1823

**Published:** 2025-05-05

**Authors:** Weiwen Xu, Yaping Chen, Weifang Yang

**Affiliations:** ^1^Department of Psychiatry, Taizhou Second Peoples’ Hospital, 317200 Taizhou, Zhejiang, China

**Keywords:** EEG biofeedback, olanzapine, schizophrenia, glycolipid metabolism, cardiac function, cognitive ability

## Abstract

**Background:**

Schizophrenia (SCZ) is a chronic mental disorder characterized by severe impairments in the daily functioning and social interactions of the patient. Compared to conventional pharmacological interventions, electroencephalogram (EEG) biofeedback offers stable and sustained effects and reduces susceptibility to relapse. This study aimed to investigate the impact of EEG biofeedback combined with olanzapine (OLZ) on glucose and lipid metabolism, cardiac function, and cognitive ability in SCZ patients.

**Methods:**

A retrospective analysis was conducted on the medical records of 66 SCZ patients who received treatment at Taizhou Second Peoples’ Hospital between June 2023 and March 2024. The patients were categorized into groups based on their treatment regimens: a single group (n = 30) and a combined therapy group (EEG biofeedback + OLZ, n = 36). Treatment efficacy and adverse reactions were compared between the groups. Key parameters assessed included glucose and lipid metabolism [total cholesterol (TC), triglyceride (TG), and fasting plasma glucose (FPG)], electrocardiographic (ECG) findings [T-wave changes, ST-segment changes, sinus bradycardia, sinus tachycardia, and other abnormalities], symptom severity [Positive and Negative Syndrome Scale (PANSS)], and cognitive function [Insight and Treatment Attitudes Questionnaire (ITAQ)] before and after treatment.

**Results:**

The total effective rate in the combined therapy group (91.67%) was significantly higher than in the single group (73.33%) (*p* < 0.05). Post-treatment, both groups exhibited significantly lower TC and FPG levels and higher TG levels compared to pre-treatment values (*p* < 0.05). However, no significant differences were observed between groups in these metabolic indices (*p* > 0.05). Similarly, no significant differences in ECG abnormalities were detected between groups, either pre- or post-treatment (*p* > 0.05). The combined therapy group demonstrated significantly greater improvements in general psychopathological symptoms, positive symptoms, negative symptoms, and PANSS scores, as well as significantly higher ITAQ scores compared to the single group (*p* < 0.05). The incidence of adverse reactions did not significantly differ between the single group (6.67%) and the combined therapy group (13.89%) (*p* > 0.05).

**Conclusion:**

EEG biofeedback combined with OLZ improves psychiatric symptoms and cognitive function of SCZ patients compared to OLZ monotherapy. Notably, the combined therapy does not exacerbate ECG abnormalities, metabolic indices, or adverse reactions, indicating a favorable safety profile.

## Introduction

Schizophrenia (SCZ) is a chronic psychiatric disorder that severely impairs the functional abilities and social interactions of the affected individuals. It manifests through abnormalities in language expression, behavior, cognitive processes, and personality traits. Key symptoms include psychotic episodes such as delusions and hallucinations, as well as cognitive impairment, social withdrawal, and diminished motivation [1, 2]. Over the course of illness, SCZ patients often experience significant mental illness and disability, which not only severely reduces their quality of life but also imposes substantial economic burdens on patients, their families, and society. Additionally, SCZ is associated with an elevated risk of comorbid conditions such as cardiovascular diseases, contributing to functional decline and reduced life expectancy [3, 4].

Globally, SCZ affects approximately 1% of the population. Between 1990 and 2019, its raw prevalence, incidence, and disability-adjusted life years increased by over 65%, 37%, and 65%, respectively, while age-standardized estimates remained stable [5]. The rising burden of SCZ has made it the focus of medical research.

Olanzapine (OLZ), a second-generation antipsychotic, is widely used in the treatment of SCZ. By antagonizing dopamine D2 and serotonin 5-hydroxytryptamine 2A receptors, OLZ effectively alleviates positive and negative symptoms of the disorder. Despite its rapid and significant therapeutic effects, the long-term efficacy of OLZ monotherapy is often suboptimal [6, 7]. On the other hand, electroencephalogram (EEG) biofeedback therapy is a non-invasive psychotherapeutic approach that uses biofeedback training to enable patients to modify cognitive, behavioral, and physiological activities. This process helps establish healthier behavior patterns and improve their social functioning. EEG is known for its stable and durable effects, with minimal risk of relapse. It has shown promise as an auxiliary treatment for conditions such as hyperkinetic syndrome, anxiety disorders, and neurasthenia [8, 9]. However, its utility as an auxiliary therapy in SCZ remains underexplored.

This study retrospectively analyzed clinical data from 66 SCZ patients who received treatment at Taizhou Second Peoples’ Hospital between June 2023 and March 2024. The study aimed to evaluate the effects of EEG biofeedback combined with OLZ on glucose and lipid metabolism, cardiac function, and cognitive abilities in SCZ patients.

## Data and Methods

### General Information

This study retrospectively analyzed the clinical data of 66 SCZ patients diagnosed with SCZ who received treatment at Taizhou Second Peoples’ Hospital between June 2023 and March 2024. The patients were divided into two groups based on their treatment regimen: the single group, which was given OLZ monotherapy (n = 30), and the combined group, which was given EEG biofeedback therapy combined with OLZ treatment (n = 36). The inclusion criteria were as follows: (1) Diagnosis of SCZ based on the International Classification of Diseases, Tenth Revision (ICD-10) [10]; (2) Availability of complete clinical data with no missing records; (3) Age between 18 and 65 years. Exclusion criteria: (1) Presence of other physical comorbidities; (2) Presence of severe medical conditions; (3) Known allergy to the study medication; (4) Poor adherence to the prescribed treatment plan.

### Methods

The single group was given OLZ monotherapy. Patients were prescribed OLZ (H20223572, Jiangsu Enhua Pharmaceutical Co., Ltd., Xuzhou, China) orally at an initial dose of 5 mg/day. The dose was gradually increased to 15–20 mg/day over two weeks, depending on the progression of the patient’s condition. Treatment continued for 3 months.

EEG biofeedback therapy was added to the OLZ treatment regimen in the combined group. EEG biofeedback therapy was performed using an EEG biofeedback instrument (20152260554, Guangzhou Runjie Medical Equipment Co., Ltd., Guangzhou, China). Electrodes were placed on the ear and scalp to monitor and analyze brainwaves, including sensory motor rhythm (SMR), β, and θ waves. Positive or negative feedback was provided based on changes in the brain power level of patients. Each treatment session lasted 20 minutes and was conducted once per day.

The EEG biofeedback therapy was administered for 12 consecutive days, followed by another 15-day interval, then repeated for another 12 days, followed by another 15-day interval. This cycle continued for a total duration of three months.

### Observation Indicators

Treatment effect evaluation: The treatment outcomes were categorized as obvious, effective, or ineffective based on the reduction rate of the Positive and Negative Syndrome Scale (PANSS) total score post-treatment. An obvious effect was defined by a reduction rate of total scores >50%. An effective outcome was characterized by a reduction rate of total scores >25% but ≤50%. An ineffective outcome was identified when the reduction rate of total score was <25%. The total treatment effectiveness was calculated as the sum of the obvious effect and effective outcomes.

Glucose and lipid metabolism indices: Fasting venous blood samples were collected from all participants before and after treatment in the morning. Samples were centrifuged at 3000 rpm for 10 minutes, and serum was collected and stored at –40 ℃ to prevent degradation. Serum total cholesterol (TC) and triglyceride (TG) were quantified using enzyme-linked immunosorbent assay (ELISA) kits (E-BC-K109-M and E-BC-K261-M, Elabscience, Wuhan, China) on an automatic biochemical analyzer (BS-280, Mindray, Yangzhou, China). Fasting plasma glucose (FPG) levels were measured using the fasting blood kinase method. The pre- and post-treatment changes in glucose and lipid metabolism were compared between the groups.

Electrocardiogram evaluation: Electrocardiograms (ECGs) were performed using the MAC 5500 electrocardiogram machine (General Electric Healthcare, Milwaukee, WI, USA) before and after treatment. Abnormal ECG findings, including T-wave changes, ST-segment changes, sinus bradycardia, and sinus tachycardia, were documented. The incidence of ECG abnormalities was compared between the two groups.

Assessment of symptom severity: Symptom severity was assessed using the Positive and Negative Syndrome Scale (PANSS) [11], which comprises three subscales: positive symptoms (7 items), negative symptoms (7 items), and general psychopathological symptoms (16 items). Each item is rated on a 7-point Linkert scale, with higher scores indicating more severe symptoms. The PANSS demonstrated excellent reliability, with Cronbach’s alpha coefficients ranging from 0.82 to 0.94.

Evaluation of cognitive function: Cognitive function was evaluated using the Insight and Treatment Attitudes Questionnaire (ITAQ) [12], which includes 11 items with a total score range of 0–22. Higher scores reflect greater self-awareness and a more positive treatment attitude.

Adverse reactions: Adverse reactions, including gastrointestinal disturbances, lethargy, weight gain, and constipation, were recorded in the two groups during the treatment period. The total incidence of adverse reactions was compared between the two groups.

### Statistical Analysis

Statistical analysis was conducted using SPSS 20.0 software (IBM, Armonk, NY, USA). The Shapiro-Wilk test was used to assess data normality. Measurement data conforming to a normal distribution were expressed as mean ± standard deviation ($\bar{x}$ ± s), with comparisons between groups conducted using independent sample *t-*tests. Paired sample *t*-tests were used to compare pre- and post-treatment within the same group. Results were expressed as [Median (P25, P75)] for data not conforming to a normal distribution, and the Mann-Whitney U test was used for between-group comparisons. Categorical data were expressed as n (%), with differences among the statistical data analyzed using the chi-square test. In the chi-square test, Pearson’s chi-square test was applied when all theoretical frequencies (T) were ≥5 and the total sample size n ≥40. When 1 ≤ T < 5 and n ≥40, Yates’s continuity correction was used. Fisher’s exact test was utilized when T <1 or n <40. A *p*-value < 0.05 was considered statistically significant.

## Results

### Comparison of Baseline Characteristics Between the Two Groups

No significant differences were observed in age, gender, education level, duration of illness, or medication history between the single and combined therapy groups (*p* > 0.05). These findings indicate that the groups were comparable at baseline (Table 1).

**Table 1.** **Baseline characteristics of the two groups [$\bar{x}$ ± s, n (%)]**.

Index		Single group (n = 30)	Combined group (n = 36)	*Z/t/χ^2^*	*p*-value
Age (years)		47.77 ± 9.18	46.72 ± 10.37	0.431	0.668
Gender	Male	20 (66.67)	24 (66.67)	0.000	1.000
	Female	10 (33.33)	12 (33.33)		
Education level	Junior high school and below	21 (70.00)	28 (77.78)	0.518	0.472
	Junior high school or above	9 (30.00)	8 (22.22)		
Disease course (month)		16.00 (9.00, 21.25)^&^	11.50 (7.25, 21.75)^&^	493.500	0.549
Medication history (years)		6.94 ± 2.19	6.38 ± 2.46	0.967	0.337

Note: ^&^Data was expressed as median (P25, P75).

### Comparison of Treatment Efficacy

The total treatment effectiveness rates were 73.33% for the single group and 91.67% for the combined group. The combined group demonstrated significantly higher treatment effectiveness compared to the single group (*p* < 0.05) (Table 2).

**Table 2.** **Comparison of treatment efficacy between the two groups [n (%)]**.

Groups	n	Obvious effect	Effective	Ineffective	Total treatment effectiveness
Single group	30	6 (20.00)	16 (53.33)	8 (26.67)	22 (73.33)
Combined group	36	13 (36.11)	20 (55.56)	3 (8.33)	33 (91.67)
χ ^2^					3.960
*p*-value					0.047

### Comparison of Glucose and Lipid Metabolism

Before treatment, there were no significant differences in TC, triglyceride (TG), or FPG levels between the two groups (*p* > 0.05). Post-treatment, TC and FPG levels were significantly reduced, while TG levels were significantly increased in both groups compared to their respective baseline values (*p* < 0.05). However, there were no significant differences in post-treatment TC, TG, or FPG levels between the two groups (*p* > 0.05) (Table 3).

**Table 3.** **Comparison of glucose and lipid metabolism between the two groups before and after treatment ($\bar{x}$ ± s)**.

Time	Groups	n	TC (mmol/L)	TG (mmol/L)	FPG (mmol/L)
Before treatment	Single group	30	4.53 ± 1.05	1.24 ± 0.31	6.23 ± 1.28
	Combined group	36	4.45 ± 1.07	1.27 ± 0.26	6.29 ± 1.34
*t-*value			0.305	0.430	0.185
*p*-value			0.761	0.669	0.854
After treatment	Single group	30	3.92 ± 0.45^*^	1.58 ± 0.36^*^	5.20 ± 1.24^*^
	Combined group	36	3.71 ± 0.48^*^	1.49 ± 0.28^*^	5.01 ± 1.37^*^
*t*-value			1.820	1.142	0.586
*p*-value			0.073	0.258	0.560

Note: Compared to before treatment, ^*^p < 0.05; TC, total cholesterol; TG, triglyceride; FPG, fasting plasma glucose.

### Comparison of Cardiac Function Indices

No significant differences in ECG abnormalities, including T-waves, ST-segment changes, sinus bradycardia, or sinus tachycardia, were observed between the two groups before and after treatment (*p* > 0.05, Tables 4,5). Additionally, no significant changes in ECG parameters were detected within either group following treatment (*p* > 0.05, Table 6).

**Table 4.** **Comparison of ECG changes between the two groups before treatment [n (%)]**.

Groups	n	T-wave change	ST-segment change	Sinus bradycardia	Sinus tachycardia	Abnormality
Single group	30	2 (6.67)	1 (3.33)	3 (10.00)	1 (3.33)	7 (23.33)
Combined group	36	5 (13.89)	1 (2.78)	0 (0.00)	1 (2.78)	7 (19.44)
χ ^2^		0.300	Fisher	1.819	Fisher	0.148
*p*-value		0.584	1.000	0.117	1.000	0.700

Note: ECG, electrocardiograph.

**Table 5.** **Comparison of ECG changes between the two groups after treatment [n (%)]**.

Groups	n	T-wave change	ST-segment change	Sinus bradycardia	Sinus tachycardia	Abnormality
Single group	30	2 (6.67)	1 (3.33)	1 (3.33)	0 (0.00)	4 (13.33)
Combined group	36	3 (8.33)	1 (2.78)	0 (0.00)	0 (0.00)	4 (11.11)
χ ^2^		0.000	Fisher	Fisher	-	0.000
*p*-value		1.000	1.000	0.455	-	1.000

Note: ECG, electrocardiograph.

**Table 6.** **Comparison of ECG changes before and after treatment within the group**.

Groups	Time	T-wave change	ST segment change	Sinus bradycardia	Sinus tachycardia	Abnormality
Single group	Before treatment	2 (6.67)	1 (3.33)	3 (10.00)	1 (3.33)	7 (23.33)
(n = 30)	After treatment	2 (6.67)	1 (3.33)	1 (3.33)	0 (0.00)	4 (13.33)
	χ ^2^	0.000	0.000	0.268	Fisher	1.002
	*p*-value	1.000	1.000	0.605	1.000	0.317
Combined group	Before treatment	5 (13.89)	1 (2.78)	0 (0.00)	1 (2.78)	7 (19.44)
(n = 36)	After treatment	3 (8.33)	1 (2.78)	0 (0.00)	0 (0.00)	4 (11.11)
	χ ^2^	0.141	0.000	-	Fisher	0.966
	*p*-value	0.708	1.000	-	1.000	0.326

Note: ECG, electrocardiograph.

### Comparison of Symptom Severity and Cognitive Function

At baseline, the two groups had no significant differences in the general psychopathological symptoms scores, positive symptoms scores, negative symptoms scores, total PANSS scores, or ITAQ scores (*p* > 0.05). After treatment, both groups demonstrated reductions in general psychopathological symptoms scores, positive symptoms scores, and total PANSS scores compared to baseline (*p* < 0.05). The combined group also showed significant reductions in negative symptom scores, whereas the single group did not (*p* > 0.05).

Post-treatment, the combined group exhibited significantly lower general psychopathological symptoms scores, positive and negative symptoms scores, and total PANSS scores compared to the single group (*p* < 0.05). Additionally, the ITAQ score was significantly higher in the combined group than in the single group (*p* < 0.05, Table 7).

**Table 7.** **Comparison of cognitive ability and attitude before and after treatment between the two groups [$\bar{x}$ ± s, Median (P25, P75)]**.

Time	Groups	n	General psychopathological symptoms score	Positive symptoms score	Negative symptoms score	Total PANSS score	ITAQ score
Before treatment	Single group	30	36.48 ± 4.11	14.57 ± 3.18	17.60 ± 6.31	68.65 ± 8.04	6 (5, 7)
	Combined group	36	35.80 ± 3.76	14.64 ± 3.15	17.44 ± 5.12	67.88 ± 7.09	6 (5, 7)
*t/Z*			0.701	0.092	0.111	0.413	539.500
*p*-value			0.486	0.927	0.912	0.681	0.995
After treatment	Single group	30	32.83 ± 3.96^*^	12.03 ± 2.62^*^	15.80 ± 4.19	60.18 ± 6.75^*^	8 (7, 9)
	Combined group	36	30.16 ± 3.57^*^	9.22 ± 1.35^*^	13.50 ± 3.96^*^	51.76 ± 5.48^*^	10 (9, 11)
*t/Z*			2.879	5.609	2.289	5.594	139.000
*p*-value			0.005	<0.001	0.025	<0.001	<0.001

Note: Compared to before treatment, ^*^p < 0.05; PANSS, Positive and Negative Syndrome Scale; ITAQ, Insight and Treatment Attitudes Questionnaire.

### Comparison of Adverse Reactions

The incidence of adverse reactions was 6.67% in the single group and 13.89% in the combined group. However, the differences were not statistically significant (*p* > 0.05, Table 8).

**Table 8.** **Comparison of adverse reactions between the two groups during treatment [n (%)]**.

Groups	n	Gastrointestinal reactions	Drowsiness	Weight gain	Constipation	Total occurrence
Single group	30	1 (3.33)	0 (0.00)	0 (0.00)	1 (3.33)	2 (6.67)
Combined group	36	2 (5.56)	1 (2.78)	1 (2.78)	1 (2.78)	5 (13.89)
χ ^2^		0.000	Fisher	Fisher	Fisher	0.300
*p*-value		1.000	1.000	1.000	1.000	0.584

## Discussion

OLZ is a commonly prescribed antipsychotic to treat SCZ. Despite its efficacy, long-term OLZ use is associated with significant side effects, such as weight gain, cardiometabolic syndrome, and diabetes, as well as limited effectiveness as a standalone treatment over extended periods [13]. Emerging evidence highlights that SCZ patients exhibit significantly reduced EEG α wave power compared to healthy individuals. These changes are notably pronounced in the occipital region and are significantly correlated with the course of disease [14, 15]. Notably, certain antipsychotic drugs modulate EEG patterns, including α waves, as part of their therapeutic mechanism, suggesting that targeting brain wave regulation could alleviate SCZ symptoms [16, 17].

EEG biofeedback therapy is effective in the treatment of depression, anxiety, hyperactivity, and sleep disorders [18, 19]. In this study, the combined treatment of OLZ and EEG biofeedback significantly improved treatment outcomes compared to OLZ alone, as evidenced by the higher total effective rate in the combined group (91.67%) versus the single group (73.33%). These findings indicate that EEG biofeedback assisted OLZ significantly affects the therapeutic efficacy. Post-treatment, the PANSS scores for general psychopathological symptoms and other dimensions decreased in both groups. Notably, the combined group exhibited lower scores for general psychopathological symptoms, negative symptoms, positive symptoms, and total PANSS compared to the single group. These findings are consistent with previous research. For example, one study demonstrated that standard rehabilitation combined with EEG biofeedback training induced significant changes in quantitative EEG and auditory event-related potential patterns in male SCZ patients, which were closely associated with improvements in positive, negative, and general symptoms [20]. Similarly, Surmeli* et al*. [21] demonstrated that quantitative EEG training significantly improved cognitive outcomes in SCZ patients undergoing rehabilitation, reducing positive and negative symptoms (measured by PANSS scores) by approximately 20%, with cognitive improvements remained stable for most participants over a 22-month follow-up period. These findings, together with our results, suggest that EEG biofeedback combined with OLZ is an effective strategy for alleviating psychiatric symptoms in SCZ patients.

Cognitive function encompasses intellectual abilities such as perception, reasoning, and memory, while cognitive impairment reflects deficits in these abilities. Cognitive impairment is a core feature of SCZ, affecting approximately 98% of patients who demonstrated significant cognitive decline compared with their pre-disease state. This decline spans multiple domains, with varying degrees of severity across different cognitive fields [22, 23]. In this study, the combined group exhibited significantly higher post-treatment ITAQ scores than the single group, highlighting the potential of EEG biofeedback in improving cognitive function and emotional well-being. These findings suggest that EEG biofeedback could serve as a valuable adjunctive therapy when combined with pharmacological interventions to provide a more comprehensive treatment regimen for SCZ.

A systematic review revealed that patients with mental illnesses undergoing pharmacotherapy experienced significant benefits from rehabilitation treatment incorporating EEG biofeedback. This biofeedback method positively influenced cognitive processes, emotional states, and anxiety levels [24]. Accumulating research highlights EEG biofeedback as an effective self-regulation technique that enables individuals to actively modulate their brain activity patterns. This capability allows direct intervention in the neural mechanisms underlying cognition and behavior [25].

OLZ improves SCZ symptoms through mechanisms such as the inhibition of 5-hydroxytryptamine 2A (5HT2A) and histamine receptors. However, it is associated with adverse effects, including increased appetite, disruption of insulin and leptin metabolism, and abnormal insulin secretion, leading to enhanced fat deposition and worsening glucose and lipid metabolism disturbances [26, 27]. In our study, no significant differences were observed in TC, TG, or FPG levels between the single and combined groups post-intervention. Additionally, the two groups had no significant difference in ECG findings or adverse reaction rates. These findings align with those of Singh *et al*. [28], who reported a high safety profile for EEG biofeedback in SCZ treatment. These results suggest that combining EEG biofeedback with OLZ does not exacerbate effects related to ECG abnormalities or metabolic disturbances compared to OLZ monotherapy, underscoring its safety in clinical application.

However, this study has several limitations, including a relatively small sample size, data collection from a single hospital, and the absence of neurofactor dynamic monitoring. Future research should address these limitations by expanding the sample size and conducting multi-center studies. Such studies would enable a more comprehensive evaluation of the combined effects of EEG biofeedback and OLZ on glucose and lipid metabolism, cardiac function, and cognitive function in SCZ patients, while further elucidating the safety and efficacy of this combined therapeutic approach.

## Conclusion

In summary, the combination of EEG biofeedback and OLZ demonstrates significant therapeutic benefits in the treatment of SCZ. This combined therapy enhances cognitive function and reduces symptom severity in SCZ patients. Furthermore, it promotes greater patient engagement with treatment while maintaining a favorable safety profile. Notably, the combined therapy does not exacerbate ECG abnormalities, glucose and lipid metabolism disturbances, or adverse reactions compared to OLZ monotherapy, supporting its potential as a safe and effective treatment strategy.

## Availability of Data and Materials

The datasets used and/or analyzed during the current study are available from the corresponding author on reasonable request.
